# Sciatic Nerve Compression after a Chronic Proximal Hamstring Tear: A Report of Two Cases and a Narrative Review of the Literature

**DOI:** 10.3390/life13081762

**Published:** 2023-08-17

**Authors:** Michael Gattringer, Georg Schalamon, Hannes Pichler, Franziska Lioba Breulmann, Heinz Buerger, Georg Mattiassich, Martin Bischofreiter

**Affiliations:** 1 Department of Orthopedic and Trauma Surgery, Clinic Diakonissen Schladming, 8970 Schladming, Austria; 2 Department of Orthopedic Surgery, Ordensklinikum Barmherzige Schwestern Linz, Vinzenzgruppe Center of Orthopedic Excellence, Teaching Hospital of the Paracelsus Medical University Salzburg, 5020 Salzburg, Austria; 3 Department of Orthopedic Sports Medicine, Klinikum Rechts der Isar, Technical University of Munich, 81675 Munich, Germany

**Keywords:** proximal hamstring tear, sciatic nerve compression, proximal hamstring avulsion, sciatica, proximal hamstring rupture

## Abstract

Proximal hamstring tears are among the most common injuries afflicting athletes and middle-aged individuals. Sciatic nerve compression after a proximal hamstring injury, which can occur due to scar formation and subsequent irritation or compression of the nerve, is an infrequent but severe complication with few cases documented in the literature. No evidence is available about the optimal treatment for sciatic nerve symptoms after proximal hamstring injuries. In this case report, we present two cases involving patients primarily treated conservatively at another institution after suffering from a proximal hamstring injury and developing sciatic nerve symptoms over the course of a few months. Both were treated with open neurolysis at our institution without reattachment of the ruptured muscles to the ischial tuberosity due to the chronicity of the injuries. Both patients exhibited neurological symptoms over two years, which recovered after surgery. These two cases show that neurolysis of the sciatic nerve without reattachment of the proximal hamstring muscles is an applicable option for the treatment of chronic proximal hamstring tears with sciatic nerve compression. Further studies will be needed to validate this hypothesis.

## 1. Introduction

A proximal hamstring tear is a common injury among athletes and physically active individuals. As a significant source of pain and functional impairment, it leads to decreased performance, absence from training sessions, and decreased competition time. The hamstring muscle group consists of three muscles originating from the ischial tuberosity: the long head of the biceps femoris, the semitendinosus, and the semimembranosus [1]. The grades of proximal hamstring injuries range from a simple strain to the complete avulsion of all three origins of the ischial tuberosity. In partial tears, the rupture typically occurs in the musculotendinous unit, whereas complete tears primarily result in a total avulsion from the ischial tuberosity [1]. The incidence of proximal hamstring tears varies widely depending on the population studied and the type of injury. Maniar et al. reported an incidence of 0.81 cases per 1000 h in field-based team sports [2]. Proximal hamstring tears are also prevalent in the general population, and this prevalence increases with age. Female patients mostly sustain injuries during daily activities, whereas male patients sustain injuries during sports [3]. The close anatomical proximity of the sciatic nerve to the ischial tuberosity (within about 1.2 cm) and the hamstring muscles can precipitate neurologic dysfunction due to the compression or stretching of the nerves caused by scar tissue, constituting a rare but severe complication of this injury [4]. The incidence of proximal hamstring tears and subsequent sciatic nerve compression has been reported to vary greatly depending on the population studied. Authors generally agree that the incidence of complete tears is higher than the avulsion of one or two muscles of the ischial tuberosity [5]. This case report describes the primarily conservative treatment of two patients suffering from acute proximal hamstring tears, which were later complicated by sciatic nerve compression, via neurolysis of the sciatic nerve conducted at our institution, as well as a narrative review of the current literature on this topic.

## 2. Case Report One

**Figure 1:**
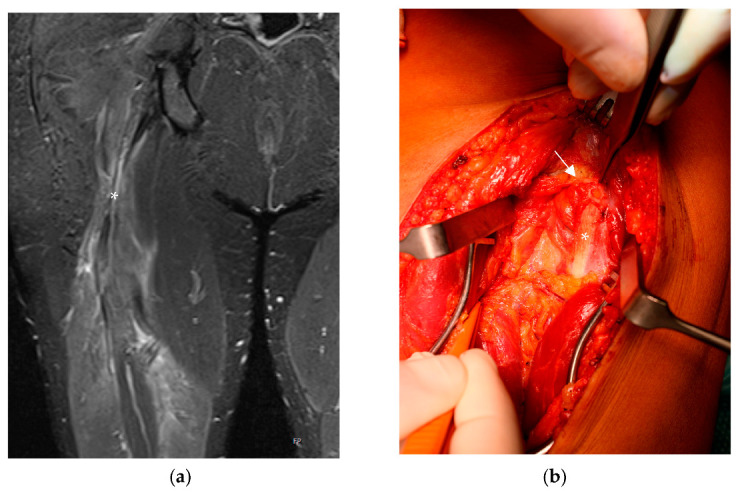
(**a**) MRI of the right thigh (coronal plane). Hematoma around the sciatic nerve (*) and retraction of the long head of the biceps femoris. (**b**) Intraoperative picture taken during neurolysis of the sciatic nerve showing the massive scar formation (→) around the entrapped nerve (*).

First, we present the case of a 38-year-old male patient who presented to our institution with prolonged posterior thigh pain and a giving way phenomenon of the right leg after a fall from 4 m at a construction site three years prior to his arrival. This patient had no relevant medical history, was healthy before his fall, and was physically active in recreational sports. He was initially treated for an additional basilar skull fracture and an epidural hematoma with persistent double vision at a level-one trauma center. Regarding the proximal hamstring injury, the patient was treated conservatively because of the single tendon rupture and his other severe and more critical injuries. In the subsequent three years, the patient approached three institutions because he could not engage in sports due to the giving way phenomenon, a strength deficit, and persistent pain while sitting. Several MRIs revealed a rupture of the biceps femoris long head and a partial rupture of the semimembranosus at the musculotendinous unit with progressive scar formation around the sciatic nerve (Figure 1a). Despite prolonged physical therapy and stationary rehab, his symptoms did not improve; so, we decided to perform surgical treatment with microscopic neurolysis of the sciatic nerve without reattachment of the biceps femoris.

The surgery was performed under general anesthesia, with the patient placed in the prone position, and without the usage of a tourniquet. A longitudinal incision was made at the border of the proximal to the middle third of the thigh with a sharp dissection of the fascia. After longitudinally incising the fascia, we found extensive scar formation around the sciatic nerve starting at the long head of the biceps femoris, as described in the MRI, with nerve compression (Figure 1a,b). The nerve was primarily dissected distally around the healthy tissue before we performed microscopic neurolysis from distal to proximal. We did not re-attach the long head of the biceps femoris due to the vital origins of the semimembranosus and semitendinosus and the retraction of the biceps femoris as a consequence of the chronicity of the injury. No drainage equipment was applied after the surgery due to exact coagulation. Postoperatively, a splint was not applied, nor was an orthosis administered, and the patient’s right lower limb was immediately immobilized. Four weeks postoperatively, the patient reported ameliorated pain and the giving way phenomenon. Furthermore, he began to engage in mild sports activity and was able to return to sports several weeks later. Two years following the operation, the patient is still free of complaints, and his sports level has returned to that before his injury.

## 3. Case Report Two

Next, we present the case of a 49-year-old male patient who slipped and fell at home and felt immediate pain in his left buttock. He was transferred to a local hospital and was diagnosed with a left hip contusion after obtaining an X-ray. Due to prolonged pain and a distinct hematoma, he visited another hospital two weeks later. He was admitted to an MRI, through which a complete tear of the proximal hamstring tendons with a retraction of over 10 cm was diagnosed. Nevertheless, the patient was not admitted to surgery and was treated conservatively with rest, ice, compression, and elevation (RICE); physical therapy; and stationary rehab. Two years later, the patient presented with persistent posterior thigh pain, a strength deficit, pain while sitting, paresthesia throughout the lateral part of the leg and the dorsum of the foot and a giving way phenomenon due to shooting pain in the left buttock. An electromyographic study of the anterior tibialis muscle and a nerve conduction study of the tibial nerve showed normal nerve and muscle function compared to the right leg.

**Figure 2:**
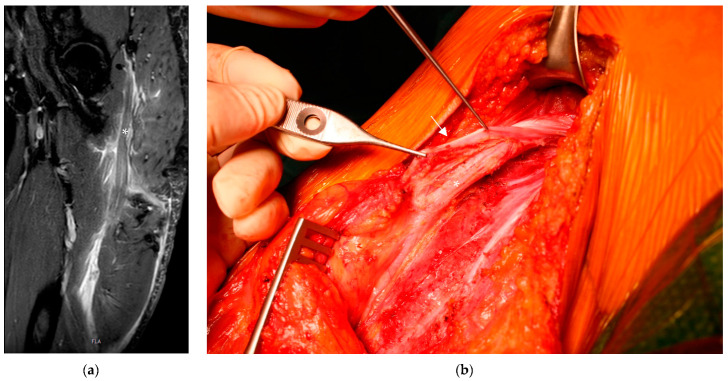
(**a**) MRI of the left thigh (in the coronal plane), which also shows an enormous hematoma around the sciatic nerve (*) and a rupture of all three muscles. (**b**) Intraoperative pictures showing the former peritendineum (→) wrapped around the sciatic nerve (*).

A recent MRI showed fatty degeneration of the hamstring muscle group and scar formation around the sciatic nerve (Figure 2a). Despite the availability of physiological nerve conduction studies, we opted for open neurolysis of the sciatic nerve since the patient presented persistent symptoms over a period of two years despite undergoing prolonged physical therapy. As in the first case, we chose to make a longitudinal incision with the patient lying in the prone position, for which a tourniquet was not used. After the dissection and incision of the fascia, the sciatic nerve was visualized and found to be stretched due to scar formation caused by the former peritendineum around the nerve, as shown in Figure 2b. Microscopic neurolysis was performed, and in this case, we reattached the biceps femoris muscle to the semimembranosus to place an additional layer over the nerve before closing the fascia to prevent further scar formation. We also chose to administer functional rehabilitation without a splint or orthosis, and physical therapy was started immediately after the surgery. Postoperatively, the giving way phenomenon and shooting pain disappeared over weeks, whereas the strength deficit, paresthesia, and posterior thigh pain while sitting persisted for as long as one year postoperatively.

## 4. Discussion

Proximal hamstring injuries are among the most common musculoskeletal injuries among athletes and the general population, accounting for 10% of all injuries in field-based team sports [2]. Due to a lack of epidemiological studies, the true incidence of complete proximal hamstring tears and sciatic nerve compression after a proximal hamstring injury still needs to be verified. Wilson et al. reported a very high rate of sciatic nerve symptoms in 27.8% of cases in their retrospective analysis of proximal hamstring tears, including radiating pain (22.2%), sensory deficits (6.8%), and motor deficits (4.9%). They also divided their cohorts into conservative and operative treatment, where the last group had a higher rate of sciatic nerve injury (24.2% vs. 32.8%). This outcome may be limited because more severe injuries (complete tears and tears with a retraction over 2 cm) were mainly treated operatively [5]. Irger et al., on the other hand, reported a rate of 5% neurologic deficits in their retrospective analysis of only surgically treated cases. They only included patients with complete avulsion injuries of the hamstring muscles [3]. Further studies will be needed to determine the true incidence of sciatic nerve damage after proximal hamstring tears and develop treatment algorithms since there are also cases with an acute onset of sciatic nerve symptoms that resolve via conservative treatment [6,7].

Several risk factors have been described for proximal hamstring injuries, including age, previous hamstring injuries, anterior cruciate ligament injuries, and previous calf strain being the most significant. Patients with a history of a prior hamstring tear are 2.7 times more likely to sustain another hamstring injury [8]. The sports disciplines with the highest rates of proximal hamstring injuries are soccer, tennis, sprinting, water skiing, running, and martial arts [3]. The typical mechanism of injury in sports is an unintentional strong hip flexion combined with an extended knee, which can occur following a rapid change of direction in running disciplines or after a loss of balance while water skiing, for example [1]. In cases of complete hamstring tears, patients usually present with pain in the posterior thigh, ecchymosis, and a palpable defect at the musculotendinous junction of the hamstring muscles. Nevertheless, some authors state that complete tears are still underdiagnosed because of a lack of awareness of the injury [9]. In one or two tendon avulsions and strain injuries, the clinical findings are less evident than those mentioned above, and the damage can easily be overlooked. Therefore, clinical examination must be performed accurately in cases of vague posterior thigh pain. The need for an MRI should be generously interpreted for all patients presenting with persistent posterior thigh pain [9,10]. Proximal hamstring injuries can be classified into acute or chronic proximal hamstring tears depending on the time of diagnosis, which is often delayed because of late consultation or a missed diagnosis. There is still no consensus about the definition of an acute injury. A proposed timeframe of under six weeks has yet to be widely accepted [11]. Wood et al. defined an MRI-based classification of a proximal hamstring tear in 2012 (Table 1), including therapy recommendations depending on the number of ruptured tendons and the grade of muscle retraction [12]. Surgical treatment is never recommended for simple muscle strains at the musculotendinous unit classified as a Type-Two injury. Surgery is recommended in cases of bony avulsions, which are primarily obtained in adolescents (Type-One injury); incomplete tears (Type-Three injuries), when the patient is still symptomatic after conservative treatment; or Type-Three injuries when the tendons are retracted over 2 cm. Complete tears are classified as Type-Four or Type-Five injuries, depending on the grade of retraction. Nevertheless, surgery is obligatory for all patients with complete hamstring tears. The presence of sciatic nerve symptoms is not included in this classification [12].

In 2015, Lempainen et al. proposed a new MRI-based treatment algorithm differentiating between one, two, and three tendon avulsions and recommending surgery depending on the patient’s age and activity level. The authors recommend early surgery for all professional athletes and other high-demand individuals for every type of tendon avulsion. Conservative treatment is recommended for cases of one tendon avulsion sustained by recreational athletes and inactive demand patients as well as for two tendon avulsions in the same patient group if the patient in question is asymptomatic. The cited authors recommend early surgery for all patients with three tendon avulsions [1]. As in the classification developed by Wood et al., sciatic nerve symptoms are not mentioned in this classification system. Conservative treatment typically includes RICE, followed by physical therapy and stretching exercises. Nonsteroidal anti-inflammatory drugs, corticosteroid injections, or shockwave therapy are not recommended for acute hamstring injuries. Also, platelet-rich plasma injections did not present any benefits in the return-to-sports period [13]. Surgical treatment typically involves reattaching the torn muscle or tendon to the ischial tuberosity using sutures or anchors and can be performed endoscopically or in an open form despite most authors favoring the open approach. Additional neurolysis of the sciatic nerve in cases of entrapment is sometimes necessary for chronic tears, but a corresponding treatment algorithm is still unavailable [14]. Several systematic reviews have reported inconsistent outcomes after the repair of acute and chronic proximal injuries. Bodendorfer et al. and Harris et al. reported significantly better outcomes in strength and overall patient satisfaction following acute repairs. In contrast, the review by van der Made et al. showed no significant differences in the parameters mentioned above between acute and delayed repair [15,16,17]. No evidence is available on the treatment of sciatic nerve compression and the timing of the corresponding surgery. In our cases, the primary goal was the neurolysis of the sciatic nerve. Furthermore, only one tendon was ruptured in the first patient, so we did not see any indication of hamstring reinsertion. We only performed open neurolysis of the nerve, which satisfied our patients, allowing them to return to sports after several weeks.

## 5. Conclusions

Sciatic nerve compression after partial and complete proximal hamstring tears is a rare but severe complication, and evidence regarding an optimal treatment is unavailable. To obtain an evidenced-based treatment algorithm, further high-quality prospective studies must be conducted on proximal hamstring injuries in general, such as the PHACT study, to determine possible risk factors that lead to the formation of extensive scar tissue and the compression of the sciatic nerve [18]. To the best of our knowledge, this is the first report about sciatic nerve symptoms after proximal hamstring injuries treated with neurolysis alone without reattachment of the hamstring tendons to the ischial tuberosity. These cases show that our procedure is applicable in chronic cases of proximal hamstring tears complicated by sciatic nerve symptoms, and reattachment of the torn muscles to the ischial tuberosity may not be necessary for every patient. Further studies are needed to investigate this hypothesis.

## Figures and Tables

**Table 1:** Wood classification of proximal hamstring injuries and operative therapies.

Type	Characteristics	Surgery Recommended
Type 1	Bone avulsion	Only if patient is symptomatic after initial conservative therapy
Type 2	Musculotendinous injury	No
Type 3	Incomplete tear	Only when tendons are retracted over 2 cm or patient is symptomatic after conservative treatment
Type 4	Complete tear (no retraction)	Yes
Type 5	Complete tear (with retraction)	Yes

## Data Availability

All data are included in the manuscript.
